# Visuospatial processing in patients with Alzheimer’s disease and cerebral amyloid angiopathy

**DOI:** 10.3389/fneur.2025.1647079

**Published:** 2025-09-08

**Authors:** Ana Sofia Costa, Milena Albrecht, Hani Ridwan, Jörg B. Schulz, Kathrin Reetz, João Pinho

**Affiliations:** ^1^Department of Neurology, University Hospital RWTH Aachen, Aachen, Germany; ^2^JARA Institute Molecular Neuroscience and Neuroimaging (INM-11), Juelich Research Center GmbH and RWTH Aachen University, Aachen, Germany; ^3^Department of Diagnostic and Interventional Neuroradiology, University Hospital RWTH Aachen, Aachen, Germany

**Keywords:** visuospatial processing, visual cortical functions, Alzheimer’s disease, cerebral amyloid angiopathy, CSF biomarkers, neuropsychological assessment

## Abstract

**Introduction:**

There is a well-established but poorly understood pathological and clinical overlap between cerebral amyloid angiopathy (CAA) and Alzheimer’s disease (AD). Some studies have suggested a posterior predominance of CAA-related lesions, but it remains unclear how well this can be captured by specific measures of low- to high-level visual cortical processing.

**Methods:**

We compared the characteristics of 30 patients with AD and/or CAA, grouped by impairment measures of low- to mid-level visual cortical processing, and explored associations with clinical characteristics, neurodegeneration biomarkers, CAA imaging features, and volumetric structural measures.

**Results:**

Twenty participants were classified as impaired on tasks of low- to mid-level visual cortical function. Impairment in these tasks was associated with performance on more complex visuoconstruction tasks, which in turn showed a correlation with structural integrity volume and cortical thickness in the occipital lobe. We found no association between impairment in low- to mid-level visual cortical functions or visuoconstruction tasks and specific measures of CAA or AD pathology.

**Discussion:**

Impairments in visuospatial functions, although reflecting structural damage in posterior brain regions, were not independently associated with markers of CAA or AD.

## Introduction

There is a known pathological and clinical overlap between cerebral amyloid angiopathy (CAA) and Alzheimer’s disease (AD) (1). At the neuropathological level, CAA is a degenerative small vessel disease (SVD) of the brain, characterized by the progressive deposition of *β*-amyloid (Aβ) –with Aβ40 being the major isoform - within the walls of cortical and leptomeningeal small arterioles, whereas Aβ depositions in AD are typically parenchymal (2). From a clinical point of view, CAA is characterized by different clinical presentations, both acute and progressive, mirroring damage caused by a wide range of processes from macro- and microbleeds and cortical superficial siderosis to wider-spread alterations in white and gray matter structure and connectivity, which are also commonly found in patients with AD (3). Given such overlaps, the clinical differentiation of cognitive impairment due to AD and/or CAA is increasingly recognized to be blurred but remains understudied. While several studies have suggested a posterior predominance of CAA-related lesions (4–6), it remains unclear how well this can be captured clinically by specific measures of visuospatial processing and how this would differ from patients with AD, which frequently also present deficits in visuospatial processing. The previous studies on this association exhibit heterogenous results. Some results indicate an association between visuospatial scores and imaging markers of CAA, such as cerebral microbleeds or white matter hyperintensities (5, 7, 8). Conversely, other studies have found no clear predominance of deficits in visuospatial processing among patients with CAA (9–11). These discrepancies seem to be influenced not only by the type of tasks used to assess visuospatial function but could also be explained by participant characteristics, particularly the concomitant presence of other pathologies like AD, which has not always been adequately controlled for in previous studies. Our objective was to examine the relationship between impairments in low- to high-level visual cortical processing and markers of CAA and AD pathology.

## Materials and methods

The data presented in this study was generated from a prospective longitudinal observational study on AD and CAA (CAADMI). All presented data was collected at baseline. Patients were eligible to participate if they were at least 50 years-old and had received a diagnosis of AD (according to the IWG criteria) (12–14) or CAA (Boston Criteria v2.0) (15), and were able to provide written informed consent. Exclusion criteria included: contraindication for MRI, other causes of intracranial hemorrhage or cortical haemosiderosis, except CAA (e.g., acquired or hereditary coagulation or platelet aggregation disorders, intracranial vascular malformations, CNS vasculitis, significant craniocerebral trauma), hereditary AD or hereditary CAA, severe cognitive impairment according to the Global Deterioration Scale > = 5 (GDS; GDS 5 = moderate dementia), and an atypical phenotypic presentation of AD (such as primary progressive aphasia or posterior cortical atrophy).

The study was approved by the local ethics committee (EK 384/20) and data protection board and is registered in the German Clinical Trials Register (DRKS00030633). All patients provided written informed consent.

### Assessment of visuospatial and other cognitive functions

Low to mid-level visuospatial functioning was assessed combining subtests from the Cortical Visual Screening Test (CORVIST) and the Leuven Perceptual Organization Screening Test (L-POST). The CORVIST (16) was developed as a screening tool to detect visual impairments in individuals with normal, normal-corrected or near-normal vision, focusing on different aspects of early visual processing by cortical centers. In this study, participants underwent the following subtests: Face Perception 1 und Face Perception 2 and Crowding. The Symbol acuity subtest was also used to record the approximate visual acuity in terms of Snellen equivalent. Face Perception 1 and 2 aim to detect impairment in the perception of faces, that might indicate right parietal lobe dysfunction or a prosopagnosia. Crowding identifies possible impairment of acuity when symbols are closely spaced. The L-POST (17–19) is a screening instrument focusing on mid-level visual processes, including figure-ground segmentation, local and global processing, shape perception and the ability to use a range of grouping cues. Each subtest uses a matching-to-sample paradigm in which one image is shown at the top and three at the bottom, and the participant must select the bottom stimulus that is most similar to the top stimulus. Participants completed all L-POST subtests except for the object recognition tasks to prevent redundancy with other measures. Performance in low to mid-level visuoperception was classified as impaired if at least three subtests in L-POST and/or at least two subtests in CORVIST (excluding the Symbol acuity subtest, as a Snellen equivalent) indicated deficits. This threshold follows available interpretation guidelines and procedures from previous studies that used the same tasks (17, 18, 20, 21), as to avoid overestimation of deficits.

We also calculated composite scores for *a priori* defined cognitive domains by averaging individual *z*-scores from measures derived from the CERAD-NAB (37, 38), the Test of Attentional Performance (TAP) (22), the Wechsler Adult Intelligence Scale (WAIS-IV), and the Rey Osterrieth Complex Figure Test (ROCFT). This process used published normative data that were adjusted for age, education, and/or sex. The cognitive domains included attention/processing speed (Trail Making Test A [TMT Part A], TAP intrinsic alertness, TAP phasic alertness), executive function (TMT Part B, phonemic fluency), language (naming, semantic fluency), memory (verbal learning, verbal recall, verbal savings, verbal recognition, nonverbal recall, nonverbal savings), and visuoconstruction (figure copy and the copy task of the ROCFT).

### Clinical parameters

Demographic, diagnostic and clinical parameters were collected from routine clinical records. Complete medical history and neurological examination was performed at study baseline. For most participants cerebrospinal fluid (CSF) neurodegeneration biomarkers had been measured at the University Medical Center Göttingen Neurochemical Laboratory in Germany using commercially available assays that have been validated in clinical populations. Given that for three participants CSF biomarkers were measured in other laboratories and cut-off values are specific to each laboratory, we also present the CSF data categorized by their pathological status (pathological or normal) based on the respective cut-off values. ApoE genotyping was conducted by the Institute for Human Genetics and Genomics at RWTH Aachen University Hospital in Germany.

### MRI protocol and measures

Patients underwent research MRI at 3-Tesla field strength (Siemens PRISMA, Erlangen, Germany) using a multimodal protocol including the following sequences (for parameters details cf. Supplementary material): isotropic high-resolution T1-weighted, T2-weighted, fluid-attenuated inversion recovery (FLAIR), and susceptibility weighted imaging (SWI).

Quantitative MRI measures were obtained using a validated open-access platform for brain image analysis (volBrain) (23). Updated images were previously anonymized, compressed and defaced using the freesurfer-based mri_deface tool. The vol2Brain (24) and lesionBrain pipelines were employed to automatically calculate the whole brain volumes, measure the thickness of cortical structures and perform segmentation of white matter lesions corrected for intracranial volume.

All MRI features assessed through visual rating were independently evaluated by two raters. This included the EPVS score for the basal ganglia and centrum semiovale, the Microbleed Anatomical Rating Scale (MARS), cortical superficial siderosis (cSS), and the multispot pattern of white matter hyperintensities. To minimize bias, the evaluation of non-haemorrhagic imaging markers of CAA was performed before rating microbleeds and cSS, with raters blinded to the patients’ characteristics.

### Statistical analysis

Descriptive statistics included counts with percentages (n [%]), mean with standard deviation (SD) or median with interquartile range (IQR), according to type of variable and data distribution. We compared demographic, clinical, and imaging characteristics and neuropsychological profiles between patients with and without low to mid-level visuoperception impairment using Mann–Whitney test or independent t-test for continuous variables and χ2 test or Fisher exact test for categorical variables. We calculated associated measures using Spearman’s correlation with correction for multiple comparisons. Statistical analyses and visualization were performed with Python programming language (version 3.12.9, with packages pandas 2.2.2, numpy 1.26.4, scipy 1.13.1., statsmodels 0.14.4, pingouin 0.5.5, seaborn 0.13.2, matplotlib 3.10.0), with a two-tailed alpha set at 0.05 as the statistical threshold for significance. An *a priori* power calculation was not feasible due to the lack of preliminary data on these specific measures and population, resulting in insufficient availability of data on the effect sizes and variability of these outcomes.

## Results

### Participants and clinical characteristics

Out of 34 participants, 2 did not have available results for the CORVIST and/or L-Post, and 2 were diagnosed with iatrogenic cerebral amyloid angiopathy. After excluding these 4 participants, the final sample consisted of 30 participants. As shown in Table 1, participants were predominantly male (*n* = 19, 63%) and had an average age of 69.6 years (SD 7.8), with a medium to high educational level (median International Standard Classification of Education level 3). The majority exhibited mild cognitive impairment (*n* = 23, 76.7%). Nine participants (30%) had probable CAA and sixteen (53%) had possible CAA (according to the Boston criteria v2.0). Twenty participants (66.7%) fulfilled the clinical-biological criteria for AD, according to the IWG criteria. Two patients fulfilled both diagnostic criteria for AD and probable CAA.

**Table 1:** Demographic and clinical characteristics in the total sample and groups of patients classified as impaired or unimpaired on low- to mid-level visuospatial functions.

Variables	Total sample *N* = 30	Low- to mid-level visuospatial functions		*p*-value
		Impaired *n* = 20	Unimpaired *n* = 10	
Age at assessment (years)	69.63 ± 7.77	70.7 ± 6.9	67.5 ± 9.1	0.427
Male sex	19 (63%)	13 (65%)	6 (60%)	0.893
Education (ISCED level)	3 (3)	2.5 (2.25)	3 (3)	0.726
Symptom duration (months)	33 (47.5)	35.5 (46.8)	29.5 (42)	0.494
Dementia in first degree relative	15 (50%)	9 (45%)	6 (60%)	1.000
Stroke in first degree relative	10 (30%)	6 (30%)	4 (40%)	0.425
Vascular risk factors or diseases				
Arterial hypertension	15 (50%)	10 (50%)	5 (50%)	1.000
Diabetes	2 (6%)	0	2 (20%)	0.124
Dyslipidaemia	16 (53%)	11 (55%)	5 (50%)	1.000
Past smoking	10 (30%)	5 (25%)	5 (50%)	0.051
Previous ischemic stroke/TIA	4 (13%)	3 (15%)	2 (20%)	1.000
Previous spontaneous ICH/SAH	3 (10%)	2 (10%)	1 (10%)	1.000
Coronary heart disease	3 (10%)	1 (5%)	1 (10%)	0.111
Atrial fibrillation	1 (3%)	0	1 (10%)	0.251
Clinical severity				
Subjective cognitive impairment	4 (13.3%)	2 (10%)	2 (20%)	0.584
Mild cognitive impairment	23 (76.7%)	17 (85%)	6 (30%)	0.181
Mild dementia	3 (10.0%)	1 (5%)	2 (20%)	0.251
ApoE status				
Homozygotic E4	3 (10%)	2 (10%)	1 (10%)	1.000
Heterozygotic E4	14 (46.6%)	6 (30%)	6 (60%)	0.724
Heterozygotic E2	2 (6%)	1 (5%)	1 (10%)	1.000
Cerebrospinal fluid				
Aβ_1-42_ (pg/mL)	454.61 ± 192.68	465.49 ± 191.10	432.85 ± 204.32	0.843
Aβ_1-42_ pathological status	20 (66.67%)	14 (70%)	6 (60%)	0.690
Aβ_1-40_ (pg/mL)^†^	10391.36 ± 3802.92	10525.72 ± 3952.22	10149.50 ± 3710.27	0.980
Aβ_1-42_/ Aβ_1-40_ ratio^†^	0.43 (0.17)	0.41 (0.17)	0.44 (0.15)	0.629
Aβ_1-42_/ Aβ_1-40_ ratio pathological status^†^	24 (85.7%)	15 (78.9%)	9 (90%)	0.632
Total tau protein (pg/mL)	538.61 (279.19)	552.0 (309.25)	375.5 (329.25)	0.367
Total tau protein pathological status	16 (52%)	13 (65%)	3 (30%)	0.121
Phosphorylated tau (pg/mL)	93.90 (41.38)	84.70 (57.36)	70.15 (74.25)	0.566
Phosphorylated tau pathological status	22 (73.3%)	15 (75%)	7 (70%)	1.000
AD diagnosis (IWG criteria)	20 (66.7%)	13 (65%)	7 (70%)	1.000
CAA (Boston criteria version 2.0)				
Possible CAA	16 (53.3%)	9 (45%)	7 (70%)	0.260
Probable CAA	9 (30%)	8 (40%)	1 (10%)	0.204

^† Data missing for n = 2.^

AD, Alzheimer’s disease; CAA, Cerebral amyloid angiopathy; ICH, intracranial cerebral hemorrhage; ISCED, International Standard Classification of Education; SAH, Subarachnoid hemorrhage; TIA, Transitory ischaemic attack.

### Performance in visuoperceptive functions and association with other measures

Twenty participants (*n* = 20) were classified as impaired in tasks of low- to mid-level visual cortical function. All participants had normal or corrected vision and there were no significant differences in the visual acuity test (Snellen equivalent) between the groups (Odds ratio [OR] = 0.43, *p* = 0.400). The study population demonstrated impaired performance in low-to-mid level visuospatial functions (Table 2) across a median of 5 subtests (IQR = 3.75; range = 0–12). There were no significant differences in demographic or clinical characteristics (Table 1)—including age, sex, education, disease duration, and clinical severity—between patients with impaired and intact low to mid-level visuospatial function. Additionally, we found no differences between the groups regarding the frequency of diagnoses of AD and/or CAA (Table 1). The two patients fulfilled both criteria for AD and CAA were categorized as impaired int. ask of low to mid-level visual cortical function.

**Table 2:** Performance in cognitive measures in the total sample and groups of patients classified as impaired or unimpaired on low- to mid-level visuospatial functions.

Variables	Total sample *N* = 30	Visuospatial functions		*p*-value
		Impaired *n* = 20	Unimpaired *n* = 10	
Montreal Cognitive Assessment, total score	22 (6)	24 (7.5)	22 (4.5)	0.299
Cognitive composite scores (*z*-scores)				
Attention	−0.97 ± 1.09	−1.15 ± 0.96	−0.64 ± 1.29	0.294
Executive function	−0.76 ± 0.91	−0.96 ± 0.76	−0.42 ± 1.09	0.188
Language	−0.57 ± 1.07	−0.77 ± 0.98	−0.21 ± 1.81	0.221
Memory	−1.25 ± 1.23	−1.39 ± 1.16	−0.98 ± 1.37	0.424
Visuoconstruction	−0.95 ± 1.11	−1.21 ± 1.04	−0.47 ± 1.10	0.095
Low to mid-level visuospatial functions				
L-POST Total score (PR)	84.6 (14.8)	81.3 (11.4)	92.0 (2.02)	0.002
L-POST Number of impaired subtests per patient	4.5 (3)	6 (2.5)	1 (2)	0.001
CORVIST ≥ 2 impaired subtests (*n*, %)	13 (46.4%)	8 (28.5%)	5 (17.8%)	0.783

CORVIST, Cortical Visual Screening Test; L-POST, Leuven Perceptual Organization Screening Test.

**Figure 1:**
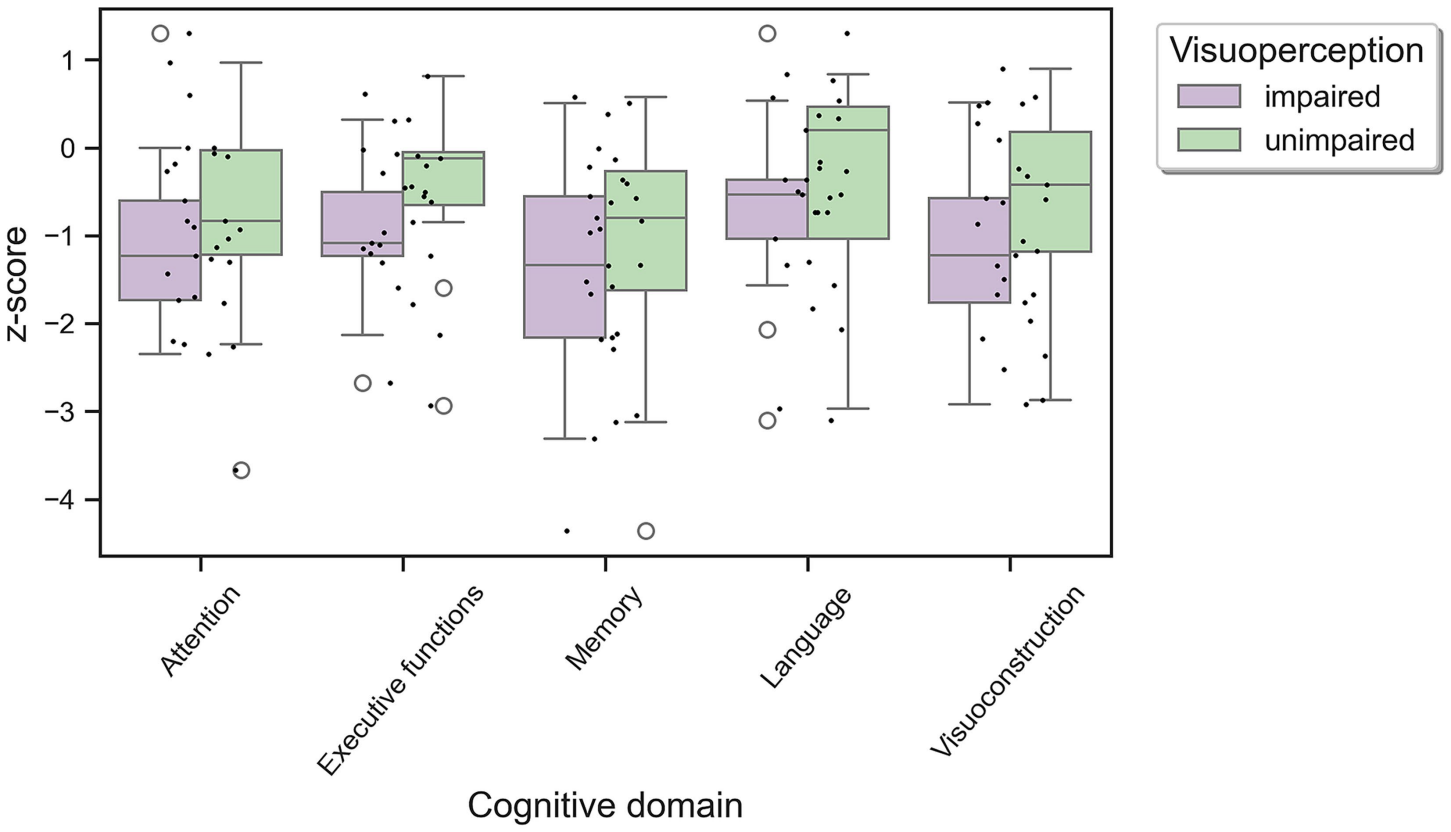
Cognitive performance of patients with Alzheimer’s disease and/or cerebral amyloid angiopathy showing either impaired or unimpaired low-to mid-level visual cortical processing. Individual data points depicted as unfilled circles represent outliers. Boxplot bars median and IQR values for each group.

As illustrated in Figure 1, while the impaired group demonstrated overall poorer performance across all cognitive domains, there were no statistically significant differences between the groups (Table 2). Imaging measures of CAA and volumetric analyses also did not reveal any differences between the groups (Table 3). Although the frequency of lobar macrohaemorrhages and convexity subarachnoid hemorrhages did not differ between impaired and unimpaired participants, interestingly macrohaemorrhages were all localized in posterior regions, predominately in parietal and occipital lobes.

**Table 3:** Magnetic resonance imaging (MRI) measures in the total sample and groups of patients classified as impaired or unimpaired on low- to mid-level visuospatial functions.

Variables	Total sample *N* = 30	Low- to mid-level visuospatial functions		*p*-value
		Impaired *n* = 20	Unimpaired *n* = 10	
CAA imaging markers				
Lobar macrohaemorrhage/cSAH	5 (16.7%)	4 (13.3%)	1 (3.3%)	0.397
Strictly lobar microbleeds (≥1)	16 (57.1%)	13 (65%)	4 (40%)	0.255
Deep microbleeds (≥1)	1 (3.7%)	0	1 (10%)	0.321
Superficial cortical siderosis	8 (28.6%)	7 (35%)	1 (10%)	0.210
Multispot WMH	21 (75%)	14 (70%)	7 (70%)	1.000
Severe CSO-EPVS*	18 (62%)	13 (68.4%)	5 (50%)	0.431
Gray matter volume (%)	44.3 (3.1)	44.4 (2.3)	44.1 (5.0)	0.832
White matter volume (%)	33.2 (1.5)	33.3 (2.0)	33.2 (1.4)	0.724
Frontal lobe volume (%)	11.4 (0.8)	11.5 (0.8)	11.1 (0.7)	0.191
Temporal lobe volume (%)	7.2 (0.7)	7.2 (0.7)	6.9 (1.1)	0.555
Parietal lobe volume (%)	6.3 (0.8)	6.3 (0.5)	6.4 (1.3)	0.464
Occipital lobe volume (%)	4.1 (0.6)	5.0 (0.5)	5.0 (0.8)	0.981
Frontal lobe cortical thickness (mm)	2.0 (0.3)	2.0 (0.3)	2.1 (0.3)	0.906
Temporal lobe cortical thickness (mm)	2.9 (0.5)	2.9 (0.3)	3.0 (0.7)	0.906
Parietal lobe cortical thickness (mm)	1.6 (0.3)	1.6 (0.3)	1.7 (0.3)	0.796
Occipital lobe cortical thickness (mm)	1.9 (0.3)	1.9 (0.4)	1.9 (0.4)	0.944

CAA, Cerebral amyloid angiopathy; cSAH, convexity subarachnoid hemorrhage; CSO-EPVS, Enlarged perivascular spaces in the centrum semiovale; WMH, White matter hyperintensities.

Gray matter, white mater and cerebral lobe volumes are presented as the percentage of total intracranial volume. * Data missing for *n* = 1 due to poor image quality.

A moderate negative association was found between impairment in low to mid-level visuospatial function and performance on more complex visuoconstruction tasks (visuoconstruction composite score: *r*_s_ = −0.55, *p* < 0.05), but no such association was observed with performance in other cognitive domains or measures of global cognition. While we did not find a significant correlation between performance in low to mid-level visuospatial function tasks and measures of structural brain integrity, such as volume and cortical thickness, we identified an association between performance in more complex visuoconstruction tasks and the volumes of the parietal (*r*_s_ = 0.46, *p* < 0.05) and occipital lobes (*r*_s_ = 0.47, *p* < 0.05). Additionally, there was a positive correlation of performance in complex visuoconstruction tasks with cortical thickness in the parietal (*r*_s_ = 0.42, *p* < 0.05) and occipital lobes (*r*_s_ = 0.44, *p* < 0.05). After correcting for multiple comparisons, only the associations with occipital lobe volume and occipital cortical thickness remained significant.

## Discussion

Despite substantial evidence from pathology and imaging studies, the clinical overlap between CAA and AD is still not well understood. Therefore, it remains a challenge to reliable differentiate between these pathologies in clinical practice. One possibility is to focus on possible spatio-temporal differences of well-described neurodegenerative or imaging markers in both disorders and use these different patterns to guide the identification of more sensitive and specific clinical markers. Following this premise, the current study aimed to assess whether the posterior predominance of CAA-related lesions could be detected through specific measures of visuospatial processing in a clinical population enriched for AD and CAA pathologies.

Our results show that performance in visuoperception (encompassing low to mid-level visuospatial cortical functions) was frequently impaired in this sample and was associated with worse performance on visuoconstruction tasks (both simple and complex copy tasks). However, only the visuoconstruction tasks showed a correlation with the structural volume and cortical thickness of the occipital lobe.

Our results are generally consistent with previous studies that have not identified deficits in measures indicative of posterior dysfunction in CAA patients, despite pathological and imaging evidence suggesting a posterior predominance of CAA pathology. For example, in a study of 77 patients with CAA with and without ICH (9), deficits in visuospatial processing (12%) were indeed less frequent than deficits in other cognitive domains, such as verbal memory (13.5%), language (26%), executive functions (37.5%) or psychomotor speed (30%). In a retrospective sample of patients with CAA from a memory clinic, we also did not found a clear pattern of cognitive performance suggestive of predominant deficits in visuoperception or visuoconstruction (10). A recent study (11) also reported no association between performance in visuospatial tasks on the Addenbrooke’s Cognitive Examination (ACE-R) and CSF markers of AD pathology, nor CAA neuroimaging markers in 35 patients with possible and probable CAA from a prospective cohort study.

Given the screening nature of both the CORVIST and L-POST, it cannot be denied that more comprehensive assessments targeting the same cognitive processes could increase specificity (25, 26). Nevertheless, their choice as measures in this study aimed to achieve reliability in the assessment deficits in low to mid-level visuospatial cortical functions, while reducing patient burden by not excessively increasing the assessment protocol duration. We also aimed to avoid overestimation of deficits by following the available interpretation recommendations, namely by declining classification at a single subtest level (18). This is particularly relevant for CORVIST, as there is less evidence on its validity and reliability compared to the L-POST. Still, it is generally assumed that any errors on CORVIST could indicate impairment (27).

We also found no association between impairment in visuospatial tasks or visuoconstruction tasks and measures of CAA or AD pathology. This result is particularly interesting given that many prior studies examining visuoperception in CAA have not accounted for possible AD pathology. This comorbidity could be particularly relevant in this situation given that the severity of CAA pathology seems to increase significantly with increased severity of AD pathology and that this increase is especially notable in the occipital region compared to other regions (4). As such, it could be expected that patients with CAA show deficits in visuospatial processing particularly when AD pathology is also present. Although at least in our group of patients we did not observe this association at a clinical level, only two patients had a clear overlap in diagnostic criteria, and both showed impaired processing in visual cortical tasks. There are other possible explanations for this lack of association with CSF biomarkers and CAA imaging markers in ours and other studies. On the one hand, the overlap between the groups may hinder the identification of specific associations, as they are also characterized by a relative range reduction within each group (for example, relative low presence of Aβ-negative patients). Conversely, as suggested by other authors, it is plausible that alternative biomarkers, including plasma biomarkers (28), may demonstrate superior discriminative validity and facilitate improved group differentiation. Similarly, advances in neuroimaging methods are promising, but the majority of automatic methods (29) are yet to be validated with regards to their discriminative value in these populations.

Differences in study design and the groups being compared (e.g., healthy controls versus case–control groups with non-CAA small vessel disease) may also account for some of the discrepancies in previous findings. While some studies utilize tasks that evaluate low-to-mid level visual cortical functions similar to the ones we employed in the current study, such as the VOSP (5) or the Benton Judgment of Line Orientation test (8), other studies relied on more complex or time-dependent tasks. The use of more complex tasks may introduce confounding factors, as patients with CAA may also exhibit impairments in other cognitive domains (7, 10, 30). Furthermore, such deficits may in some cases not be fully accounted for CAA or be influenced by other (co)-pathologies. The same argument applies to using time-dependent tasks to assess visuospatial function. Given that patients with CAA often exhibit deficits in processing speed, which is also demonstrated in our current study, this may act as an important confounder. To control for some of these potential confounders, we employed tasks that are not time-dependent and were specifically designed to assess low to mid-level cortical visual function. Additionally, we chose to define the groups based on the presence of impairment in these tasks to avoid circularity, considering the frequent overlap between CAA and AD (1, 31). In designing the study to better understand the differential roles of the two conditions, we believe it is more beneficial to focus on participants with clear evidence of either pathology or both, as this approach more accurately reflects the challenges encountered in clinical practice. However, including participants without evidence of AD and/or CAA in future studies could help to account for other factors contributing to alterations in visual processing, such as aging (32).

Previous studies that identified an association between visuospatial impairment in patients with CAA and damage to posterior regions primarily mainly relied on measures of tract-based spatial statistics and tractography to assess white matter integrity (5, 6) as well some network-based approaches using diffusion tensor imaging (33). While we found an association between performance on more complex visual tasks and occipital volume and cortical thickness automatic measures, our analyses did not incorporate measures of white matter integrity. Being a limitation of the current study, we cannot rule out the possibility that this type of measures may be more sensitive to microdamage associated with CAA and AD, particularly in early disease stages. Still, other studies have suggested that cortical thinning may play a significant role as it partially mediates associations between typical haemorrhagic imaging markers of CAA and cognitive impairment (34). Taken together, this underscores the need for more specific imaging methods to obtain a more nuanced understanding of these relationships that we aim to apply in future analyses using the longitudinal data arising from this prospective study. More advance metrics using diffusion tensor imaging, as for example peak width of skeletonized mean diffusivity (35), could not only be more sensitive to early and more subtle dysfunction but help answer if the changes in the white matter integrity are a result from Wallerian degeneration or a direct product of the association neuropathological processes in the white matter regions.

As the current cross-sectional study design does not allow for this, using longitudinal data could also help clarify possible causal relationships between pathophysiological changes depicted by biomarkers and imaging markers, and changes in visuospatial processing. Another limitation of the current study is the small sample size, which may reduce statistical power. This could, for example, limit the detection of subtle differences between the groups or associations between variables that, in this case, may even partially overlap. Conversely, as the sample is derived from a prospective study we provided a comprehensive sample characterization, including CSF neurodegeneration biomarkers and imaging data, along with detailed neuropsychological assessment. This comprehensive approach enables a thorough phenotyping of a clinically representative yet heterogeneous patient cohort. Future studies should ideally aim to achieve this level of characterization with larger samples to allow for more precise analyses.

In summary, our results indicate that impairment of low- to mid-level visuospatial functions, although reflecting structural damage in posterior brain regions, is not independently associated with markers of CAA or AD. Therefore, it should not be used as the sole clinical indicator of a specific underlying pathology. These results should motivate further research to address the challenge of defining a specific cognitive profile for CAA patients, given the frequent overlap of co-pathologies, particularly AD. This is especially important for patients who present with cognitive decline as the first manifestation of CAA (36).

## Data Availability

The datasets presented in this article are not readily available because of data sharing restrictions imposed by the informed consent and current privacy and data protection legislations. Requests to access the datasets should be directed to acosta@ukaachen.de.
