# Supplementary material for: Visuospatial processing in patients with Alzheimer’s disease and cerebral amyloid angiopathy
Source: Front Neurol. 2025 Sep 8;16:1647079. doi: 10.3389/fneur.2025.1647079 (PMC12450703; doi:10.3389/fneur.2025.1647079)
Supplement: Supplementary file 1 [file Table_1.DOCX]

**Supplementary Table 1**: Magnetic resonance imaging parameters at a 3T Siemens PRISMA scanner

| **Sequence** | **Characteristics** |
| --- | --- |
| T1 MP-RAGE GRAPPA | TR = 2400 ms, TE = 2.36 ms, slice thickness 0.8 mm, matrix 224x224, resolution 288 |
| T2-weighted imaging | TR = 4430 ms, TE = 94 ms, range of slice thickness 3.0–4.0 mm, range of voxel size = 0.35–1.03 mm^3^ |
| Fluid attenuated inversion recovery (FLAIR) | TR = 9000 ms, TE = 90 ms, matrix=256x256; slices=42, flip°=150 |
| Susceptibility weighted imaging (SWI) | TR = 28 ms, TE = 20 ms, matrix=256x256, flip°=15 |
